# Approximating distance between sets by multivalued coupling with application to uniformly convex Banach spaces

**DOI:** 10.1186/s13660-018-1720-0

**Published:** 2018-06-11

**Authors:** Binayak S. Choudhury, Pranati Maity, Nikhilesh Metiya, Mihai Postolache

**Affiliations:** 1 Department of Mathematics, Indian Institute of Engineering Science and Technology, Howrah, India; 2 Department of Mathematics, National Institute of Technology, Rourkela, India; 3 Department of Mathematics, Sovarani Memorial College, Howrah, India; 4 China Medical University, Taichung, Taiwan; 5 Institute of Mathematical Statistics and Applied Mathematics, Romanian Academy, Bucharest, Romania; 6 Department of Mathematics and Computer Science, University Politehnica of Bucharest, Bucharest, Romania

**Keywords:** 54H10, 54H25, 47H10, Metric spaces, Coupling, Best proximity pair, Optimal approximate solution, Uniformly convex Banach space

## Abstract

In this paper, our aim is to ascertain the distance between two sets iteratively in two simultaneous ways with the help of a multivalued coupling define for this purpose. We define the best proximity points of such couplings that realize the distance between two sets. Our main theorem is deduced in metric spaces. As an application, we obtain the corresponding results in uniformly convex Banach spaces using the geometry of the space. We discuss two examples.

## Introduction

In this paper, our intention is to discuss a procedure for finding the distance between two given subsets of a metric space by using a multivalued coupling between two sets. The aforesaid coupling is a multivalued coupled cyclic type mapping between two sets we define. There are several works for determining the minimum distance mentioned, where nonselfmappings, both cyclic and noncyclic, have been utilized. Such problems are known as best proximity point problems for the concern mappings and were introduced by Eldered et al. [16] in 2006. This line of research developed through works like [1, 6, 7, 9, 12–14, 23, 24, 27, 30, 31] in subsequent times. Multivalued mappings have been considered in fixed and proximity point problems in works like [2, 8, 18, 20, 26]. Essentially, this is a kind of global optimization problem, which, in the approach mentioned, is treated as the problem of finding an optimal approximate solution of a fixed point equation (or a fixed point inclusion in the case of a multivalued mappings) although the exact solution may not exist, which is actually the case where the two sets are disjoint. This situation is of interest for the present problem. Within the above framework, we take a new approach to this problem by defining a multivalued coupling between two sets. We define a multivalued coupled proximity point for these couplings, which simultaneously realize two best proximity pairs between two sets. We discuss the existence of such points under a contractive condition. We apply our theorem to obtain the corresponding result in uniformly convex Banach spaces. Uniformly convex Banach spaces constitute a category of Banach spaces including Hilbert spaces and in which interest has been widespread. We use some bit of Banach space geometry to obtain our result. Further, we illustrate our results with examples. Open problems are discussed at the end of the paper.

## Mathematical preliminaries

Throughout this paper, $(X, d)$ stands for a general metric space. Let *A* and *B* be two subsets of *X*. A pair of points $(a, b)$ with $a\in A$ and $b\in B$ is said to be a best proximity pair if $d(a, b)=d(A, B)$ where $d(A, B)= \operatorname{inf} \{d(x, y): x\in A \text{ and } y\in B\} $. If $T: A \longrightarrow B$ is a nonselfmapping for which there exists $z\in A$ such that $d(z, Tz)= d(A, B)$, that is, where $(z, Tz)$ is a best proximity pair, we say that *z* is a best proximity point of *T*, and the problem of finding such a point is known as the best proximity point problem. It is a global optimization problem in that it seeks to find a point $z\in A$ that minimizes the value of $d(z, Tz)$ over $z \in A$ subject to the condition that the minimum is $d(A, B)$. On the other hand, it is an extension of the fixed point problem and reduces to that problem in the special case where $A\cap B\neq\emptyset$. The following are the concepts from set-valued analysis which we use in this paper. We consider the following classes of subsets of the metric space *X*:

$$\begin{aligned} &N(X) = \{ A: A \text{ is a nonempty subset of } X\}, \\ &B(X) = \{ A: A \text{ is a nonempty bounded subset of } X\}, \end{aligned}$$

and

$$ CB(X) = \{A: A \text{ is a nonempty closed and bounded subset of } X\}. $$

For $A, B \in B(X)$, the functions *D* and *δ* are defined as follows:

$$\begin{aligned} &d(A, B) = D(A, B)= \operatorname{inf} \bigl\{ d(a, b): a\in A, b\in B\bigr\} , \\ &\delta(A, B)= \operatorname{sup} \bigl\{ d(a, b): a\in A, b\in B\bigr\} . \end{aligned}$$

If $A= \{a\}$, then we write $D(A, B)= D(a, B)$ and $\delta(A, B)= \delta(a, B)$. In addition, if $B= \{b\}$, then $D(A, B)= d(a, b)$ and $\delta(A, B)= d(a, b)$. Obviously, $D(A, B)\leq\delta(A, B)$. For all $A, B, C\in B(X)$, the definition of $\delta(A, B)$ yields the following:

$\delta(A, B)= \delta(B, A)$, $\delta(A, B)\leq\delta(A, C) + \delta(C, B)$, $\delta(A, B)= 0$ iff $A = B = \{a\}$, $\delta (A, A)= \operatorname{diam} A$ [17].

The *δ*-distance has all the properties of a metric except one. It has been used in works like [4, 5, 10]. We use this concept in our theorem.

Let *A* and *B* be two nonempty subsets of a metric space $(X, d)$, and let $T:A \longrightarrow CB(B)$ be a multivalued mapping. A point $x^{\ast}\in A$ is called a best proximity point of *T* if $D(x^{\ast }, Tx^{\ast})= \operatorname{inf} \{d(x^{\ast}, y): y\in Tx^{\ast}\}= \operatorname{dist}(A, B)$ [2]. This is a natural generalization of its single-valued counterpart described above. A fixed point *x* of a multivalued mapping *T* is given by the inclusion relation $x\in Tx$. Now there need not be a fixed point of the multivalued mapping in general. Here the task in the best proximity point problem is to find a global minimum of the function $x\mapsto D(x, Tx)$ by constraining an approximate solution of the inclusion relation $x\in Tx$ to satisfy $D(x, Tx)= \operatorname{dist}(A, B)$.

Next, we give the following:

### Definition 2.1

Let $(X, d)$ be a metric space, and let *A*, *B* be two nonempty subsets of *X*. A mapping $F\colon X\times X \longrightarrow X$ is said to be a coupling with respect to *A* and *B* if $F(x, y)\in B$ for $(x, y)\in A \times B$ and $F(x, y)\in A $ for $(x, y)\in B \times A$.

The following definition is a multivalued generalization of Definition 2.1.

### Definition 2.2

Let $(X, d)$ be a metric space, and let *A*, *B* be two nonempty subsets of *X*. A multivalued mapping $F\colon X\times X\longrightarrow N(X)$ is said to be a multivalued coupling with respect to *A* and *B* if $F(x, y)\subseteq B $ for $(x, y)\in A \times B$ and $F(x, y)\subseteq A $ for $(x, y)\in B \times A$.

Our purpose here is to realize the minimum distance between two sets *A* and *B* through a coupled best proximity point for coupling, which we define further.

### Definition 2.3

Let $(X, d)$ be a metric space, and let *A*, *B* be two nonempty subsets of *X*. Let $F\colon X\times X\longrightarrow X$ be a coupling with respect to *A* and *B*. An element $(x, y)\in A \times B$ is called a coupled best proximity point if $d(x, F(x, y))=d(A, B)$ and $d(y, F(y, x))=d(A, B)$, where $d(A, B)= \inf \{d(x, y): x \in A \mbox{ and } y \in B\}$.

### Definition 2.4

Let $(X, d)$ be a metric space, and let *A*, *B* be two nonempty subsets of *X*. Let $F\colon X\times X\longrightarrow N(X)$ be a multivalued coupling with respect to *A* and *B*. An element $(x, y)\in A \times B$ is called a coupled best proximity point if $D(x, F(x, y))=d(A, B)$ and $D(y, F(y, x))=d(A, B)$, where $d(A, B)= \inf \{d(x, y): x \in A\mbox{ and }y \in B\}$.

Note that proximity points for coupled mapping have been defined in [21]. Our definition is for both single-valued and multivalued couplings, which is different from the above-mentioned concept. Also, the present work is in a different course from that of [21].

Here the problem of finding coupled best proximity points for multivalued couplings is viewed as that of finding simultaneous optimal approximate solutions of the coupled fixed point inclusions $x \in F(x, y)$ and $y \in F(y, x)$ for a multivalued coupling *F* in the set $A\times B$ such that the solution satisfies $D(x, F(x, y))= D(y, F(y, x))=d(A, B)$. In general, the exact solutions may not exist. This is surely the case where $d(A, B)\neq0$, which is of interest here. From another viewpoint, it is the problem of simultaneous minimization of $D(x, F(x, y))$ and $D(y, F(y, x))$ for $x\in A$, $y\in B$ such that the minimum values at the point of optimality are the global minimum $d(A, B)$. It is to be noted that the pairs $(x, F(x, y))$ and $(y, F(y, x))$ are in general different. Thus the distance between two sets is obtained through two different pairs of points, that is, in the process, we obtain two best proximity pairs. Also, the coupled best proximity point may be not unique. The following illustration elucidates these points.

### Example 2.1

Let $X= {\mathbb {R}}^{2}$ with the metric *d* defined as $d((x_{1}, y_{1}), (x_{2}, y_{2}))= \vert x_{1} - x_{2} \vert + \vert y_{1} - y_{2} \vert $. Let $A=\{0\}\times\mathbb {R}$ and $B=\{1\}\times\mathbb {R}$. Then *A* and *B* are nonempty subsets of *X*, and $d(A, B)=1$. Consider $F\colon X\times X \longrightarrow N(X)$ defined as

$$ F(x,y)= \textstyle\begin{cases} \{1\}\times[1, p] &\mbox{if } x=(0, p)\in A \mbox{ and } y=(1, q)\in B, \\ \{0\}\times[0, \frac{q}{2}] &\mbox{if } x=(1, q)\in B\mbox{ and } y=(0, p)\in A, \\ \{2, 3\} &\mbox{otherwise}. \end{cases} $$

Here *F* is a coupling with respect to *A* and *B*. For any $c\neq0$, the pair $((0, c), (1, 0)) \in A \times B$ is a coupled best proximity point of *F*.

We discuss an application of our main result in uniformly convex Banach spaces.

### Definition 2.5

([3])

A Banach space *X* is said to be uniformly convex if for every *ϵ* satisfying $0 < \epsilon\leq2$, there is $\delta(\epsilon )> 0$ such that, for all $x, y \in X$,

$$ \Vert x \Vert = \Vert y \Vert = 1 \quad\text{and} \quad \Vert x-y \Vert \geq \epsilon\quad\Longrightarrow \quad \biggl\Vert \frac {x+y}{2} \biggr\Vert \leq1- \delta(\epsilon). $$

We use the following results in our application.

### Lemma 2.1

([16])

*Let* *A* *be a nonempty closed and convex subset*, *and let* *B* *be a nonempty closed subset of a uniformly convex Banach space*. *Let* $\{x_{n}\} $ *and* $\{z_{n}\}$ *be sequences in* *A*, *and let* $\{y_{n}\}$ *be a sequence in* *B* *satisfying*:

(i) $\Vert z_{n}-y_{n} \Vert \longrightarrow d(A, B)$ *as* $n \longrightarrow\infty$,
(ii) *for every* $\epsilon> 0$, *there exists* $N_{0}$ *such that* $\Vert x_{m}-y_{n} \Vert \leq d(A, B)+ \epsilon$ *for all* $m > n \geq N_{0}$.

*Then*, *for every* $\epsilon> 0$, *there exists* $N_{1}$ *such that* $\Vert x_{m}-z_{n} \Vert \leq\epsilon$ *for all* $m > n \geq N_{1}$.

### Lemma 2.2

([16])

*Let* *A* *be a nonempty closed and convex subset*, *and let* *B* *be a nonempty closed subset of a uniformly convex Banach space*. *Let* $\{x_{n}\} $ *and* $\{z_{n}\}$ *be sequences in* *A*, *and let* $\{y_{n}\}$ *be a sequence in* *B* *satisfying*:

(i) $\Vert x_{n}-y_{n} \Vert \longrightarrow d(A, B)$ *as* $n \longrightarrow\infty$,
(ii) $\Vert z_{n}-y_{n} \Vert \longrightarrow d(A, B)$ *as* $n \longrightarrow\infty$.

*Then* $\Vert x_{n}-z_{n} \Vert \longrightarrow0$ *as* $n \longrightarrow\infty$.

Lemma 2.1 and Lemma 2.2 from [16] are generalized in the general case of a function modular space [25] as far as any uniformly convex Banach space is a function modular space generated by a function modular *ρ* that satisfies $(UC1)$ and has $\Delta_{2}$-property. Therefore, Lemma 2.1 and Lemma 2.2 from [16] are particular cases of the results from [22] and [32] enriching the knowledge on the geometry of function modular spaces.

## Main results

### Theorem 3.1

*Let* *A* *and* *B* *be two nonempty closed subsets of a complete metric space* $(X, d)$. *Let* $F\colon X\times X\longrightarrow B(X)$ *be a coupling with respect to* *A* *and* *B* *satisfying the following inequality*:

3.1 $$ \delta\bigl(F(x, y), F(u, v)\bigr)\leq\frac{k}{2} \bigl[d(x, u) + d(y, v) \bigr]+ (1-k) d(A, B), $$

*where* $x, v\in A$, $y, u\in B$, *and* $k\in(0, 1)$.

1. *Then there exist two sequences* $\{x_{n}\}$ *and* $\{y_{n}\}$ *in* *A* *and* *B*, *respectively*, *such that*
  $$ \underset{n \longrightarrow\infty}{\lim}d(x_{n}, y_{n+1}) = d(A, B)\quad \textit{and}\quad \underset{n \longrightarrow\infty}{\lim }d(y_{n}, x_{n+1}) = d(A, B). $$
2. *Further*, *if* $\{x_{n}\}$ *and* $\{y_{n}\}$ *are Cauchy sequences*, *then* *F* *has a coupled best proximity point*.

### Proof

Starting with arbitrary $x_{0}\in A$ and $y_{0}\in B$, we construct two sequences $\{x_{n}\}$ and $\{y_{n}\}$ in *X* such that

3.2 $$ x_{n+1} \in F(y_{n}, x_{n}) \quad\text{and}\quad y_{n+1} \in F(x_{n}, y_{n}) \quad\text{for all } n\geq0. $$

Then $\{x_{n}\}$ is a sequence in *A*, and $\{y_{n}\}$ is a sequence in *B*. By (3.1) and (3.2) we have

3.3 $$\begin{aligned} d(x_{1}, y_{2})&\leq\delta \bigl(x_{1}, F(x_{1}, y_{1})\bigr)\leq\delta \bigl(F(y_{0}, x_{0}), F(x_{1}, y_{1}) \bigr) \\ &= \delta\bigl(F(x_{1}, y_{1}), F(y_{0}, x_{0})\bigr) \\ &\leq \frac{k}{2} \bigl[d(y_{0}, x_{1})+ d(x_{0}, y_{1}) \bigr]+ (1-k) d(A, B). \end{aligned}$$

By (3.1) and (3.2) we have

3.4 $$\begin{aligned} d(y_{1}, x_{2})&\leq\delta \bigl(y_{1}, F(y_{1}, x_{1})\bigr) \\ &\leq \delta\bigl(F(x_{0}, y_{0}), F(y_{1}, x_{1})\bigr) \\ &\leq \frac{k}{2} \bigl[d(x_{0}, y_{1})+ d(y_{0}, x_{1}) \bigr]+ (1-k) d(A, B). \end{aligned}$$

From (3.3) and (3.4) we have

3.5 $$ \frac{d(x_{1}, y_{2}) + d(y_{1}, x_{2})}{2}\leq\frac{k}{2} \bigl[d(x_{0}, y_{1}) + d(y_{0}, x_{1}) \bigr]+ (1-k) d(A, B). $$

By (3.1), (3.2), and (3.5) we have

3.6 $$\begin{aligned} d(x_{2}, y_{3})&\leq \delta \bigl(x_{2}, F(x_{2}, y_{2})\bigr)\leq\delta \bigl(F(y_{1}, x_{1}), F(x_{2}, y_{2}) \bigr) \\ &= \delta\bigl(F(x_{2}, y_{2}), F(y_{1}, x_{1})\bigr) \\ &\leq \frac{k}{2} \bigl[d(y_{1}, x_{2}) + d(x_{1}, y_{2}) \bigr]+ (1-k) d(A, B) \\ &= k \biggl[\frac{d(y_{1}, x_{2}) + d(x_{1}, y_{2})}{2} \biggr]+ (1-k) d(A, B) \\ &\leq k \biggl[\frac{k}{2} \bigl[d(x_{0}, y_{1}) + d(y_{0}, x_{1}) \bigr]+ (1-k) d(A, B) \biggr]+ (1-k) d(A, B) \\ &=\frac{k^{2}}{2} \bigl[d(x_{0}, y_{1}) + d(y_{0}, x_{1}) \bigr]+ \bigl(1-k^{2}\bigr) d(A, B). \end{aligned}$$

Similarly,

3.7 $$ d(y_{2}, x_{3}) \leq\delta \bigl(y_{2}, F(y_{2}, x_{2})\bigr)\leq \frac {k^{2}}{2} \bigl[d(x_{0}, y_{1}) + d(y_{0}, x_{1}) \bigr]+ \bigl(1-k^{2}\bigr) d(A, B). $$

Let us suppose that, for some integer $n=p$,

3.8 $$ d(x_{p}, y_{p+1})\leq\frac{k^{p}}{2} \bigl[d(x_{0}, y_{1}) + d(y_{0}, x_{1}) \bigr]+ \bigl(1-k^{p}\bigr) d(A, B) $$

and

3.9 $$ d(y_{p}, x_{p+1})\leq\frac{k^{p}}{2} \bigl[d(x_{0}, y_{1}) + d(y_{0}, x_{1}) \bigr]+ \bigl(1-k^{p}\bigr) d(A, B). $$

From (3.8) and (3.9) we have

3.10 $$ \frac{d(x_{p}, y_{p+1}) + d(y_{p}, x_{p+1})}{2}\leq\frac {k^{p}}{2} \bigl[d(x_{0}, y_{1}) + d(y_{0}, x_{1}) \bigr]+ \bigl(1-k^{p}\bigr) d(A, B). $$

By (3.1), (3.2), and (3.10) we obtain

$$\begin{aligned} d(x_{p+1}, y_{p+2})&\leq \delta\bigl(x_{p+1}, F(x_{p+1}, y_{p+1})\bigr)\leq \delta\bigl(F(y_{p}, x_{p}), F(x_{p+1}, y_{p+1})\bigr) \\ &= \delta\bigl(F(x_{p+1}, y_{p+1}), F(y_{p}, x_{p})\bigr) \\ &\leq \frac{k}{2} \bigl[d(y_{p}, x_{p+1}) + d(x_{p}, y_{p+1}) \bigr]+ (1-k) d(A, B) \\ &= k \biggl[\frac{d(y_{p}, x_{p+1}) + d(x_{p}, y_{p+1})}{2} \biggr]+ (1-k) d(A, B) \\ &\leq k \biggl[\frac{k^{p}}{2} \bigl[d(x_{0}, y_{1}) + d(y_{0}, x_{1}) \bigr]+ \bigl(1-k^{p}\bigr) d(A, B) \biggr]+ (1-k) d(A, B) \\ &\leq \frac{k^{p+1}}{2} \bigl[d(x_{0}, y_{1}) + d(y_{0}, x_{1}) \bigr]+ \bigl(1-k^{p+1}\bigr) d(A, B). \end{aligned}$$

Similarly, by (3.1), (3.2), and (3.10) we obtain

$$\begin{aligned} d(y_{p+1}, x_{p+2})&\leq \delta\bigl(y_{p+1}, F(y_{p+1}, x_{p+1})\bigr) \\ &\leq \delta\bigl(F(x_{p}, y_{p}), F(y_{p+1}, x_{p+1})\bigr) \\ &\leq \frac{k}{2} \bigl[d(x_{p}, y_{p+1}) + d(y_{p}, x_{p+1}) \bigr]+ (1-k) d(A, B) \\ &= k \biggl[\frac{d(x_{p}, y_{p+1}) + d(y_{p}, x_{p+1})}{2} \biggr]+ (1-k) d(A, B) \\ &\leq k \biggl[\frac{k^{p}}{2} \bigl[d(x_{0}, y_{1}) + d(y_{0}, x_{1}) \bigr]+ \bigl(1-k^{p}\bigr) d(A, B) \biggr]+ (1-k) d(A, B) \\ &\leq \frac{k^{p+1}}{2} \bigl[d(x_{0}, y_{1})+d(y_{0}, x_{1}) \bigr]+ \bigl(1-k^{p+1}\bigr) d(A, B). \end{aligned}$$

Therefore, by induction theorem we can write that, for all $n \geq1$,

3.11 $$ d(x_{n}, y_{n+1})\leq\frac{k^{n}}{2} \bigl[d(x_{0}, y_{1}) + d(y_{0}, x_{1}) \bigr]+ \bigl(1-k^{n}\bigr) d(A, B) $$

and

3.12 $$ d(y_{n}, x_{n+1})\leq\frac{k^{n}}{2} \bigl[d(x_{0}, y_{1}) + d(y_{0}, x_{1}) \bigr]+ \bigl(1-k^{n}\bigr) d(A, B). $$

Since $k \in(0, 1)$, taking the limits as $n \longrightarrow\infty$ in these two inequalities, we have

3.13 $$ \underset{n \longrightarrow\infty}{\lim} d(x_{n}, y_{n+1}) = d(A, B) $$

and

3.14 $$ \underset{n \longrightarrow\infty}{\lim}d(y_{n}, x_{n+1})= d(A, B). $$

Further, suppose that $\{x_{n}\}$ and $\{y_{n}\}$ are Cauchy sequences. Here *X* is complete, and *A* and *B* are closed subsets of *X*. Since $\{x_{n}\}$ and $\{y_{n}\}$ are sequences respectively in *A* and *B*, there exist $x \in A$ and $y \in B$ such that

3.15 $$ x_{n}\longrightarrow x \quad\text{and}\quad y_{n} \longrightarrow y \quad\text{as } n \longrightarrow\infty. $$

By (3.13), (3.14), and (3.15) we have

3.16 $$ \underset{n \longrightarrow\infty}{\lim} d(x_{n}, y_{n+1}) = d(x, y) = d(A, B) $$

and

3.17 $$ \underset{n \longrightarrow\infty}{\lim}d(y_{n}, x_{n+1})= d(y, x) = d(x, y) = d(A, B). $$

Using (3.1), (3.2), and (3.16) we obtain

$$\begin{aligned} d(A, B)&\leq D\bigl(x, F(x, y)\bigr)\leq\delta\bigl(x, F(x, y)\bigr) \leq d(x, x_{n+1})+ \delta\bigl(x_{n+1}, F(x, y)\bigr) \\ &\leq d(x, x_{n+1})+ \delta\bigl(F(y_{n}, x_{n}), F(x, y)\bigr) \\ &= d(x, x_{n+1})+ \delta\bigl(F(x, y), F(y_{n}, x_{n})\bigr) \\ &\leq d(x, x_{n+1})+ \frac{k}{2} \bigl[d(y_{n}, x)+ d(x_{n}, y) \bigr]+ (1-k) d(A, B) \\ &\leq d(x, x_{n+1})+ \frac{k}{2} \bigl[d(y_{n}, y)+ d(y, x)+ d(x_{n}, x) + d(x, y) \bigr]+ (1-k) d(A, B) \\ &= d(x, x_{n+1})+ \frac{k}{2} \bigl[d(y_{n}, y)+ d(A, B) + d(x_{n}, x)+d(A, B)\bigr]+ (1-k) d(A, B). \end{aligned}$$

Taking the limit as $n \longrightarrow\infty$ in this inequality and using (3.15), we have

$$ d(A, B)\leq D\bigl(x, F(x, y)\bigr)\leq k d(A, B)+(1-k) d(A, B) = d(A, B), $$

which implies that $D(x, F(x, y)) = d(A, B)$. Similarly, we can prove that $D(y, F(y, x)) = d(A, B)$. Hence $D(x, F(x, y)) = d(A, B)$ and $D(y, F(y, x)) = d(A, B)$, that is, $(x, y)$ is a coupled best proximity point of *F*. □

### Remark 3.1

It is a natural question to ask under what conditions the two sequences $\{x_{n}\}$ and $\{y_{n}\}$ in *X* should converge. We give an answer to this question in the context of uniformly convex Banach spaces for the single-valued case. It is also possible to deduce a similar result by using UC property [31] in a metric space. We prefer to obtain it in uniformly convex Banach spaces since UC property is a consequence of the structure of the space for certain pairs of subsets.

### Remark 3.2

If $A \cap B \neq\emptyset$, then we obtain a multivalued coupled fixed point result for couplings. There are several recent works in which some coupled fixed point results are obtained [11, 15, 28, 29]. Further, it has been pointed out in [19] that several generalizations of existing results in fixed point theory are not actual generalizations. The fixed point result obtained under the conditions mentioned above is not a direct generalization of any existing result. Since multivalued couplings of the above type have not appeared earlier in the literature and therefore is not relevant to the discussion presented in [19].

### Example 3.1

Let $X= R^{2}$ with the metric *d* defined as $d((x_{1}, y_{1}), (x_{2}, y_{2}))= \vert x_{1} - x_{2} \vert + \vert y_{1} - y_{2} \vert $. Let $A=\{0\}\times[0, \infty)$ and $B=\{1\}\times(-\infty, 0]$. Then *A* and *B* are nonempty closed subsets of *X*, and $d(A, B)=1$. Let $F\colon X\times X \longrightarrow B(X)$ be defined as

$$ F(x, y)= \textstyle\begin{cases} \{1\}\times[-\frac{a-b}{4}, 0]& \mbox{if } x=(0, a)\in A \mbox{ and } y=(1, b)\in B,\\ \{0\}\times[0,-\frac{b-a}{4}]& \mbox{if } x=(1, b)\in B \mbox{ and } y=(0, a)\in A,\\ \{2, 2\} & \mbox{otherwise}. \end{cases} $$

Let $x, v \in A$ and $y, u \in B$. Then $x= (0, a_{1})$, $v= (0, a_{2})$, $y= (1, b_{1})$, and $u= (1, b_{2})$ for some $a_{1}, a_{2}\geq0$ and $b_{1}, b_{2}\leq0$. Then

$$\begin{aligned} & \delta\bigl(F(x, y), F(u, v)\bigr)\\ &\quad= 1+ \biggl\vert -\frac{b_{2}- a_{2}}{4}+ \frac {a_{1}- b_{1}}{4} \biggr\vert = 1+ \frac{(a_{1}+a_{2})-(b_{1}+b_{2})}{4}, \\ & \frac{k}{2} \bigl[d(x, u)+ d(y, v) \bigr]+ (1-k) d(A, B)\\ &\quad= \frac {k}{2} \bigl[1+(a_{1}-b_{2})+1+(a_{2}-b_{1}) \bigr]+(1-k) \\ &\quad= 1+ k\frac{(a_{1}+a_{2})-(b_{1}+b_{2})}{2}. \end{aligned}$$

Since $(a_{1}+a_{2})\geq0$, $(b_{1}+b_{2})\leq0$, it follows that inequality (3.1) holds for all $\frac{1}{2} \leq k < 1$.

For any $x_{0} = (0, a)\in A$ and $y_{0} = (1, b)\in B$, consider $x_{n}= (0, \frac{a-b}{4^{n}})$ and $y_{n} = (1, -\frac{a-b}{4^{n}})$. Then the sequences $\{x_{n}\}$ and $\{y_{n}\}$ satisfy (1) and (2) of Theorem 3.1. Then, applying Theorem 3.1, we conclude that $((0, 0), (1, 0))$ is a coupled best proximity point of *F*.

## Application to uniformly convex Banach spaces

In this section, we discuss the implications of our result in the previous section in uniformly convex Banach spaces for the case of single-valued couplings.

### Theorem 4.1

*Let* *A* *and* *B* *be two nonempty closed subsets of a Banach space* *X*. *Let* $F\colon X\times X\longrightarrow X$ *be a coupling with respect to* *A* *and* *B* *satisfying the following inequality*:

4.1 $$ \bigl\Vert F(x, y)- F(u, v) \bigr\Vert \leq\frac{k}{2} \bigl[ \Vert x- u \Vert + \Vert y- v \Vert \bigr]+ (1-k) d(A, B), $$

*where* $x, v\in A$, $y, u\in B$, *and* $k\in(0, 1)$.

1. *Then*, *for arbitrary* $(x_{0}, y_{0})\in A \times B$, *the sequences* $\{x_{n}\}$ *and* $\{y_{n}\}$ *respectively in* *A* *and* *B* *constructed as*
  4.2 $$ x_{n+1} = F(y_{n}, x_{n}) \quad\textit{and}\quad y_{n+1} = F(x_{n}, y_{n})\quad \textit {for all } n\geq0 $$
  *satisfy*
  $$ \underset{n\longrightarrow\infty}{\lim} \Vert x_{n}- y_{n+1} \Vert = d(A, B) \quad\textit{and}\quad \underset{n \longrightarrow\infty}{\lim} \Vert y_{n}- x_{n+1} \Vert = d(A, B). $$
2. *Further*, *if* $\{x_{n}\}$ *and* $\{y_{n}\}$ *are Cauchy sequences*, *then* *F* *has a coupled best proximity point*.

### Proof

We know that, for every $x\in X$, $\{x\}\in B(X)$. We define the mapping $T\colon X\times X \longrightarrow B(X)$ as $T(x, y) = \{F(x, y)\} \text{ for } x, y \in X$. Then all the conditions of the theorem reduce to the conditions of Theorem 3.1, and hence by Theorem 3.1, we have the results in (1) and (2) of the theorem. □

### Remark 4.1

It is a natural question to ask for circumstances under which both the sequences $\{x_{n}\}$ and $\{y_{n}\}$ converge so that Theorem 4.1 is applicable. An answer is given further by showing, by an application of Lemma 2.2, that this is the case if the coupling is between two closed and convex subsets of a uniformly convex Banach space. In general, Lemma 2.2 is not satisfied in a metric space. If the result of the lemma is valid with respect to subsets *A* and *B* in a metric space, then $(A, B)$ is said to satisfy the UC-property with respect to the pair $(A, B)$. The UC-property was introduce by Suzuki et al. [31]. We do not use this property in metric spaces; the lemma is rather used for an application in uniformly convex Banach spaces. So we omit any further reference of the UC-property.

### Lemma 4.1

*Let* $F\colon X\times X \longrightarrow X$, *where* *X* *is a normed linear space*, *be a coupling with respect to two subsets* *A* *and* *B* *of* *X*. *Suppose that* *F* *satisfies* (4.1). *Then*, *for arbitrary* $(x_{0}, y_{0})\in A \times B$, *the sequences* $\{x_{n}\}$ *and* $\{y_{n}\}$ *constructed as in* (4.2) *satisfy* $\underset{n \longrightarrow \infty}{\lim} \Vert x_{n}-y_{n} \Vert \longrightarrow d(A, B)$.

### Proof

It is clear from (4.2) that $\{x_{n}\}$ and $\{y_{n}\}$ are sequences in *A* and *B*, respectively. Using (4.1) and (4.2), for $n\geq1$, we have

4.3 $$\begin{aligned} \Vert x_{n} - y_{n} \Vert &= \bigl\Vert F(y_{n-1}, x_{n-1}) - F(x_{n-1}, y_{n-1}) \bigr\Vert \\ &= \bigl\Vert F(x_{n-1}, y_{n-1})) - F(y_{n-1}, x_{n-1}) \bigr\Vert \\ &\leq\frac{k}{2} \bigl[ \Vert y_{n-1}- x_{n-1} \Vert + \Vert x_{n-1}- y_{n-1} \Vert \bigr]+ (1-k) d(A, B) \\ &= k \Vert x_{n-1}- y_{n-1} \Vert + (1-k) d(A, B). \end{aligned}$$

By repeated application of (4.3) we get

4.4 $$\begin{aligned} \Vert x_{n} - y_{n} \Vert &\leq k \Vert x_{n-1}- y_{n-1} \Vert + (1-k) d(A, B) \\ &\leq k \bigl[k \Vert x_{n-2}- y_{n-2} \Vert + (1-k) d(A, B) \bigr]+ (1-k) d(A, B) \\ &= k^{2} \Vert x_{n-2}- y_{n-2} \Vert + \bigl(1-k^{2}\bigr) d(A, B) \\ &\leq k^{2} \bigl[k \Vert x_{n-3}- y_{n-3} \Vert + (1-k) d(A, B) \bigr]+ \bigl(1-k^{2}\bigr) d(A, B) \\ &= k^{3} \Vert x_{n-3}- y_{n-3} \Vert + \bigl(1-k^{3}\bigr) d(A, B) \\ &\cdots \\ &= k^{n} \Vert x_{0}- y_{0} \Vert + \bigl(1-k^{n}\bigr) d(A, B). \end{aligned}$$

Since $\{x_{n}\}$ and $\{y_{n}\}$ are sequences in *A* and *B*, respectively, we have

4.5 $$ d(A, B) \leq \Vert x_{n}-y_{n} \Vert \quad\text{for all } n \geq1. $$

It follows from (4.4) and (4.5) that

$$\begin{aligned} d(A, B)\leq \Vert x_{n} - y_{n} \Vert \leq k^{n} \Vert x_{0}- y_{0} \Vert + \bigl(1-k^{n}\bigr) d(A, B). \end{aligned}$$

Since $k\in(0, 1)$, taking the limit as $n \longrightarrow\infty$ in this inequality, we obtain $\underset{n \longrightarrow\infty}{\lim } \Vert x_{n}-y_{n} \Vert = d(A, B)$. □

### Remark 4.2

Lemma 4.1 is also generally valid in metric spaces. It is not necessary to assume *X* to be a normed linear space. We use this result only in the theorem for uniformly convex Banach spaces. For this reason, in particular, we assume that *X* is a normed linear space.

### Theorem 4.2

*Let* *A* *and* *B* *be two nonempty closed and convex subsets of a uniformly convex Banach space*, *and let* $F\colon X\times X \longrightarrow X$ *be a coupling with respect to* *A* *and* *B*. *Suppose that* *F* *satisfies* (4.1). *Then* *F* *has a coupled best proximity point*.

### Proof

Let $x_{0}\in A$ and $y_{0}\in B$. We construct two sequences $\{x_{n}\}$ and $\{y_{n}\}$ respectively in *A* and *B* that satisfy (4.2), that is,

$$ x_{n+1} = F(y_{n}, x_{n}) \quad\text{and}\quad y_{n+1} = F(x_{n}, y_{n}) \quad\text{for all } n\geq0. $$

By Theorem 4.1 we have

$$ \underset{n \longrightarrow\infty}{\lim} \Vert x_{n}- y_{n+1} \Vert = d(A, B) \quad\text{and}\quad \underset{n \longrightarrow\infty}{\lim} \Vert y_{n+1}-x_{n+2} \Vert = \Vert x_{n+2}-y_{n+1} \Vert = d(A, B). $$

By Lemma 2.2 we have

4.6 $$ \underset{n \longrightarrow\infty}{\lim} \Vert x_{n}- x_{n+2} \Vert = 0. $$

Again, by Theorem 4.1 we have

$$ \underset{n \longrightarrow\infty}{\lim} \Vert y_{n}- x_{n+1} \Vert = d(A, B) \quad\text{and}\quad \underset{n \longrightarrow\infty}{\lim} \Vert x_{n+1}-y_{n+2} \Vert = \Vert y_{n+2}-x_{n+1} \Vert = d(A, B). $$

By Lemma 2.2 we have

4.7 $$ \underset{n \longrightarrow\infty}{\lim} \Vert y_{n}- y_{n+2} \Vert = 0. $$

Next, we prove that $\{x_{2n}\}$, $\{y_{2n}\}$, $\{x_{2n+1}\}$, and $\{ y_{2n+1}\}$ are Cauchy sequences. We further consider the case of the sequences $\{x_{2n}\}$ and $\{y_{2n}\}$. From (4.6) and (4.7) we have that

4.8 $$ \underset{n \longrightarrow\infty}{\lim} \Vert x_{2n}-x_{2n+2} \Vert = \underset{n \longrightarrow\infty}{ \lim } \Vert y_{2n}-y_{2n+2} \Vert = 0. $$

We first establish that, given $\epsilon>0$, we can find a positive integer *N* such that, for all $m, n > N$,

4.9 $$ \operatorname{max} \bigl\{ \Vert x_{2m}- y_{2n} \Vert , \Vert y_{2m}- x_{2n} \Vert \bigr\} \leq d(A, B)+ \epsilon. $$

In view of Lemma 4.1, we can find a positive integer $p_{0}$ such that, for all $n\geq p_{0}$,

$$ \Vert x_{2n}- y_{2n} \Vert < d(A, B)+ \epsilon. $$

If (4.9) is not valid, then for some $\epsilon>0$, in view of this inequality, for all $p\geq p_{0}$, there exist $n(p)> m(p)> p$ for which

$$ \operatorname{max} \bigl\{ \Vert x_{2m(p)}- y_{2n(p)} \Vert , \Vert y_{2m(p)}- x_{2n(p)} \Vert \bigr\} \geq d(A, B)+ \epsilon $$

and

$$ \max \bigl\{ \Vert x_{2m(p)}- y_{2n(p)-2} \Vert , \Vert y_{2m(p)}- x_{2n(p)-2} \Vert \bigr\} < d(A, B)+ \epsilon. $$

Then

$$\begin{aligned} d(A, B)+ \epsilon\leq{}&\max \bigl\{ \Vert x_{2m(p)}- y_{2n(p)} \Vert , \Vert y_{2m(p)}- x_{2n(p)} \Vert \bigr\} \\ \leq{}&\max \bigl\{ \Vert x_{2m(p)}- y_{2n(p)-2} \Vert + \Vert y_{2n(p)-2}- y_{2n(p)} \Vert , \\ & \Vert y_{2m(p)}- x_{2n(p)-2} \Vert + \Vert x_{2n(p)-2}- x_{2n(p)} \Vert \bigr\} \\ < {}&d(A, B)+ \epsilon+ \max \bigl\{ \Vert y_{2n(p)-2}- y_{2n(p)} \Vert , \Vert x_{2n(p)-2}- x_{2n(p)} \Vert \bigr\} . \end{aligned}$$

Taking the limit as $p\longrightarrow\infty$ and using (4.8), we obtain

4.10 $$ \underset{p\longrightarrow\infty}{\lim}\max \bigl\{ \Vert x_{2m(p)}- y_{2n(p)} \Vert , \Vert y_{2m(p)}- x_{2n(p)} \Vert \bigr\} = d(A, B)+ \epsilon. $$

Again, for all $p\geq p_{0}$,

$$\begin{aligned} & \max \bigl\{ \Vert x_{2m(p)+2}- y_{2n(p)+2} \Vert , \Vert y_{2m(p)+2}- x_{2n(p)+2} \Vert \bigr\} \\ &\quad\leq\max \bigl\{ \Vert x_{2m(p)+2}- x_{2m(p)} \Vert + \Vert x_{2m(p)}-y_{2n(p)} \Vert + \Vert y_{2n(p)}-y_{2n(p)+2} \Vert , \\ &\qquad \Vert y_{2m(p)+2}- y_{2m(p)} \Vert + \Vert y_{2m(p)}- x_{2n(p)} \Vert + \Vert x_{2n(p)}-x_{2n(p)+2} \Vert \bigr\} \\ &\quad\leq\max \bigl\{ \Vert x_{2m(p)}-y_{2n(p)} \Vert , \Vert x_{2n(p)}-y_{2m(p)} \Vert \bigr\} \\ &\qquad {}+\max \bigl\{ \Vert x_{2m(p)+2}- x_{2m(p)} \Vert + \Vert y_{2n(p)}-y_{2n(p)+2} \Vert , \\ &\qquad \Vert y_{2m(p)+2}- y_{2m(p)} \Vert + \Vert x_{2n(p)}-x_{2n(p)+2} \Vert \bigr\} \end{aligned}$$

and

$$\begin{aligned} & \max \bigl\{ \Vert x_{2m(p)}-y_{2n(p)} \Vert , \Vert x_{2n(p)}-y_{2m(p)} \Vert \bigr\} \\ &\quad\leq\max \bigl\{ \Vert x_{2m(p)}-x_{2m(p)+2} \Vert + \Vert x_{2m(p)+2}-y_{2n(p)+2} \Vert + \Vert y_{2n(p)+2}-y_{2n(p)} \Vert , \\ &\qquad \Vert x_{2n(p)}-x_{2n(p)+2} \Vert + \Vert x_{2n(p)+2}-y_{2m(p)+2} \Vert + \Vert y_{2m(p)+2}-y_{2m(p)} \Vert \bigr\} \\ &\quad\leq\max \bigl\{ \Vert x_{2m(p)+2}-y_{2n(p)+2} \Vert , \Vert x_{2n(p)+2}-y_{2m(p)+2} \Vert \bigr\} \\ &\qquad{} +\max \bigl\{ \Vert x_{2m(p)}-x_{2m(p)+2} \Vert + \Vert y_{2n(p)+2}-y_{2n(p)} \Vert , \\ &\qquad \Vert x_{2n(p)}-x_{2n(p)+2} \Vert + \Vert y_{2m(p)+2}-y_{2m(p)} \Vert \bigr\} . \end{aligned}$$

Taking the limit as $p \longrightarrow\infty$ in the last two inequalities and using (4.8) and (4.10), we conclude that

4.11 $$ \underset{p \longrightarrow\infty}{\lim} \max \bigl\{ \Vert x_{2m(p)+2}- y_{2n(p)+2} \Vert , \Vert y_{2m(p)+2}- x_{2n(p)+2} \Vert \bigr\} = d(A, B)+ \epsilon. $$

Again,

$$\begin{aligned} & \max \bigl\{ \Vert x_{2m(p)+2}- y_{2n(p)+2} \Vert , \Vert y_{2m(p)+2}- x_{2n(p)+2} \Vert \bigr\} \\ &\quad= \max \bigl\{ \bigl\Vert F(y_{2m(p)+1}, x_{2m(p)+1})- F(x_{2n(p)+1}, y_{2n(p)+1}) \bigr\Vert , \\ &\qquad \bigl\Vert F(x_{2m(p)+1}, y_{2m(p)+1})- F(y_{2n(p)+1}, x_{2n(p)+1}) \bigr\Vert \bigr\} \\ &\quad \leq\max \biggl\{ \frac{k}{2}\bigl( \Vert y_{2m(p)+1}- x_{2n(p)+1} \Vert + \Vert x_{2m(p)+1}- y_{2n(p)+1} \Vert \bigr)+ (1-k)d(A, B), \\ &\qquad\frac{k}{2}\bigl( \Vert x_{2m(p)+1}- y_{2n(p)+1} \Vert + \Vert y_{2m(p)+1}- x_{2n(p)+1} \Vert \bigr)+ (1-k)d(A, B) \biggr\} \\ &\quad= \frac{k}{2} \bigl( \Vert y_{2m(p)+1}- x_{2n(p)+1} \Vert + \Vert x_{2m(p)+1}- y_{2n(p)+1} \Vert \bigr)+ (1-k)d(A, B) \\ &\quad= \frac{k}{2} \bigl( \bigl\Vert F(x_{2m(p)}, y_{2m(p)})- F(y_{2n(p)}, x_{2n(p)}) \bigr\Vert \\ &\qquad+ \bigl\Vert F(y_{2m(p)}, x_{2m(p)})- F(x_{2n(p)}, y_{2n(p)}) \bigr\Vert \bigr)+(1-k)d(A, B) \\ &\quad= \frac{k}{2} \biggl[\frac{k}{2} \bigl( \Vert x_{2m(p)}- y_{2n(p)} \Vert + \Vert y_{2m(p)}- x_{2n(p)} \Vert \bigr)+ (1-k)d(A, B) \\ &\qquad{}+\frac{k}{2} \bigl( \Vert y_{2m(p)}- x_{2n(p)} \Vert + \Vert x_{2m(p)}- y_{2n(p)} \Vert \bigr)+ (1-k)d(A, B) \biggr]+ (1-k)d(A, B) \\ &\quad= \frac{k}{2} \bigl[k \bigl( \Vert x_{2m(p)}- y_{2n(p)} \Vert + \Vert y_{2m(p)}- x_{2n(p)} \Vert \bigr)+ 2(1-k)d(A, B) \bigr]+ (1-k)d(A, B) \\ &\quad=\frac{k^{2}}{2} \bigl( \Vert x_{2m(p)}- y_{2n(p)} \Vert + \Vert y_{2m(p)}- x_{2n(p)} \Vert \bigr)+ (1-k) k d(A, B) + (1-k)d(A, B) \\ &\quad=\frac{k^{2}}{2} \max \bigl\{ \Vert x_{2m(p)}- y_{2n(p)} \Vert , \Vert y_{2m(p)}- x_{2n(p)} \Vert \bigr\} + \bigl(1-k^{2}\bigr)d(A, B). \end{aligned}$$

Taking the limit as $p \longrightarrow\infty$ in this inequality, we have

$$\begin{aligned} d(A, B)+ \epsilon&\leq\frac{k^{2}}{2} \bigl[d(A, B)+ \epsilon \bigr]+ \bigl(1-k^{2}\bigr)d(A, B) \\ &\leq \biggl(1-\frac{k^{2}}{2} \biggr)d(A, B)+ \frac{\epsilon k^{2}}{2} \\ &\leq d(A, B)+ \epsilon k^{2}. \end{aligned}$$

Since $0 < k < 1$, it is a contradiction. Therefore, (4.9) holds. Combining this fact with Lemma 4.1, by Lemma 2.1 we conclude that $\{x_{2n}\}$ and $\{y_{2n}\}$ are a Cauchy sequences in *A* and *B*, respectively. The sets *A* and *B* being closed, there exist $x\in A$ and $y\in B$ such that

4.12 $$ x_{2n}\longrightarrow x \quad\text{and}\quad y_{2n} \longrightarrow y \quad\text{as } n \longrightarrow\infty. $$

Proceeding exactly as in the previous case, we have that there exist $\overline{x}\in A$ and $\overline{y}\in B$ for which

4.13 $$ x_{2n+1}\longrightarrow\overline{x} \quad\text{and}\quad y_{2n+1}\longrightarrow\overline{y} \quad\text{as } n \longrightarrow \infty. $$

Then using Lemma 4.1, (4.12), and (4.13), we conclude that

4.14 $$ \Vert x - y \Vert = \Vert \overline{x} - \overline {y} \Vert =d(A, B). $$

Again, using Theorem 4.1(1) with (4.12) and (4.13), we conclude that

4.15 $$ \Vert x - \overline{y} \Vert = \Vert y - \overline {x} \Vert =d(A, B). $$

Using Lemma 2.2 with (4.14) and (4.15), we conclude that

$$ x= \overline{x} \quad\text{and}\quad y = \overline{y}. $$

From (4.12) and (4.13) it follows that

$$ x_{n}\longrightarrow x\in A \quad\text{and} \quad y_{n}\longrightarrow y\in B \quad\text{as } n \longrightarrow\infty. $$

Applying Theorem 4.1, we can prove that $d(A, B)= \Vert x - F(x, y) \Vert = \Vert y - F(y, x) \Vert $, that is, $(x, y)$ is a coupled best proximity of *F*. □

### Example 4.1

Let us consider $X= \mathbb {R}$ and $\Vert x \Vert = \vert x \vert $ for $x\in\mathbb {R}$. If $A= (-\infty, -1]$ and $B= [1, -\infty)$, then *A* and *B* are closed convex subsets of *X*. Let $F\colon X\times X\rightarrow X$ be defined as

$$ F(x, y)= \textstyle\begin{cases} -\frac{x-y}{2}& \mbox{if } x\in A \mbox{ and } y\in B,\\ \frac{y-x}{2}& \mbox{if } x\in B \mbox{ and } y\in A. \end{cases} $$

Then inequality (4.1) is satisfied. Here, *X* being a uniformly convex Banach space, we can apply Theorem 4.2 to conclude that there is a coupled proximity point of *F*.

## Conclusion

In Sect. 4, we have shown that the coupling has a coupled best proximity point in uniformly convex Banach spaces automatically by the properties of the space. Particularly, the result is valid in Hilbert spaces. It remains to be investigated under what other conditions the coupled best proximity points exist for such couplings. Also, a feature of the present work is that the results are obtained without any continuity assumption on the coupling.

Regarding error estimates, the first such results were obtained for cyclic contraction map in [33] and for coupled best proximity points for cyclic contractive mappings in [21]. It may also be possible to obtain error estimates for coupled best proximity points of coupling through the iterated sequence (4.2) in Theorem 4.1 by following the ideas of [21, 33]. Such problems of error estimation arising out of the considerations of couplings as in the present case can be taken up in future works as open problems.
